# Accurate Intraoperative Estimation of Tip-Apex Distance in the Cephalomedullary Fixation of Proximal Femoral Fractures

**DOI:** 10.7759/cureus.81508

**Published:** 2025-03-31

**Authors:** Haydar A Al Hussainy, Andre Martins, Giang Le

**Affiliations:** 1 Trauma and Orthopaedics, Northampton General Hospital, Northampton, GBR

**Keywords:** neck of femur fracture, surgical tip, tad, tfna, tip-apex distance

## Abstract

Cephalomedullary nailing systems, including the Trochanteric Femoral Nailing-ADVANCED™ (TFNA) (DePuy Synthes, Raynham, Massachusetts, United States), are widely utilised for stabilising proximal femoral fractures. A pivotal aspect of the procedure involves ensuring adequate tip-apex distance (TAD) of the lag screw below 25 mm, a measure that substantially diminishes the cutout rate.

We introduce a simple method to accurately estimate the intraoperative TAD in TFNA fixation. This technique relies on the known diameter of the shaft of the screw (the root diameter), just proximal to the threaded section.

The new technique is simple, easy, and effective in potentially reducing operative time and improving the accuracy of estimating TAD during cephalomedullary hip fracture stabilisation surgery.

## Introduction

Cephalomedullary nailing systems, such as the Trochanteric Femoral Nailing-ADVANCED™ (TFNA) (DePuy Synthes, Raynham, Massachusetts, United States), are widely used for stabilising proximal femoral fractures [1].

A key consideration in this procedure is maintaining a tip-apex distance (TAD) of less than 25 mm, as this significantly reduces the risk of screw cutout [2,3]. Screw cutout can be devastating to patients as it will potentially hinder the stability of the neck of femur fracture fixation and damage the ipsilateral hip joint.

Accurately measuring TAD intraoperatively can be challenging, particularly in the absence of a ruler while scrubbed and when relying on fluoroscopic images, which are often magnified. The previous methods involve a complex mathematical equation that depends on rough visual estimation, making it susceptible to inaccuracies due to image distortion from magnification, underexposure, overexposure, and potential malrotation [2,4].

Aim  

This report aims to introduce a simple method for accurately estimating the intraoperative TAD without needing a ruler in the cephalomedullary fixation of proximal femoral fractures. 

## Technical report

**Figure 1:**
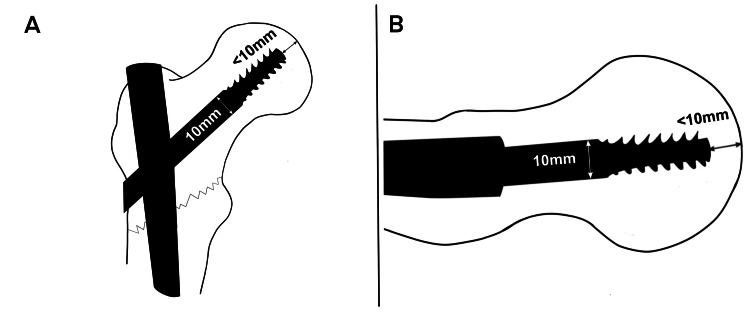
Determining TAD in an instant without needing a ruler Artwork illustration depicting a proximal femoral fracture stabilised with a cephalomedullary TFNA device. A shows the AP view, while B presents the lateral view. The TAD can be accurately measured by visually referencing to the diameter of the lag screw shaft Image Credit: Mrs. Sara Al Hussainy from the National Physical Laboratory (NPL), London, the United Kingdom TAD: tip-apex distance; TFNA: Trochanteric Femoral Nailing-ADVANCED™; AP: anteroposterior

During hip fracture fixation surgery, once the TFNA guidewire has been properly positioned within the subchondral bone at the centre of the femoral head in both the anteroposterior (AP) and lateral views, the TFNA lag screw is advanced until it is approximately 10 mm from the centre of the tip of the screw to the apex of the articular surface dome of the femoral head. This distance can now be accurately referenced to the shaft diameter of the screw, just proximal to the threaded section, which measures approximately 10 mm (precisely 10.35 mm) [5] (Figure 1).

As a result, the combined AP and lateral TAD will be consistently maintained at an optimal measurement of just below 20 mm. This simplified approach allows for a straightforward combined TAD calculation of 10+10=20, eliminating the need for complex corrected TAD formulas, ratio adjustments, or percentage-based calculations previously advocated [2,4]. Additionally, this technique is applicable to helical blades, as they share the same shaft geometry design [5]. 

## Discussion

This rapid TAD calculation technique is inspired by methods described in the literature on dynamic hip screw (DHS) fixation, particularly the DHS TAD eyeballing technique [6] (Table 1).

**Table 1:** TAD referencing to screw root diameter Lag screw particulars for a variety of commonly used cephalomedullary nails as compared to the lag screw of the DHS implant. TFNA: Trochanteric Femoral Nailing-ADVANCED™; DHS: dynamic hip screw; IMHS: intramedullary hip screw; PFN: proximal femoral nail

Type of nail/screw	Screw root diameter (mm)	Screw thread diameter (mm)	Manufacturer
TFNA	10.35	10.35	Dupuy Synthes (Raynham, Massachusetts, United States)
DHS	8	12.5	Dupuy Synthes (Raynham, Massachusetts, United States)
Gamma nail	10.5	10.5	Stryker (Kalamazoo, Michigan, United States)
IMHS	9	12.7	Smith & Nephew (London, United Kingdom)
Trigen Intertan	11	11	Smith & Nephew (London, United Kingdom)
Targon PFN	7	10.4	Aesculap (Center Valley, Pennsylvania, United States)

The proposed method relies on measuring the root diameter of the lag screw rather than its outer thread diameter. We believe the root diameter provides a more consistent reference, as it is solid rather than wavy like the threads, making it less susceptible to optical illusion errors and easier to measure accurately. 

Additionally, using a TAD reference of less than 10 mm, when measured against the root diameter, is more practical than the conventional threshold of over 12 mm for the thread diameter of most nailing screws. This method consistently results in a combined TAD of approximately 20 mm, rather than the traditional 25 mm, thereby further reducing the risk of ending up with a larger TAD and ultimately leading to the potential screw cutout. Moreover, this approach simplifies intraoperative calculations, easing the cognitive burden on surgeons during procedures. 

This technique is adaptable to other cephalomedullary nailing systems, provided the root diameter of the lag screw is known and predetermined [7-11] (Table 1).

Notably, the method has no inherent limitations; however, going less than or more than a TAD of 20 mm will remain a decision for the operating surgeon to make with high TAD accuracy, regardless of the underlying condition or the preoperative bony ailments. The authors have applied and verified this TAD measurement technique in numerous patients with remarkable accuracy. Its implementation has the potential to reduce operative time, minimise radiation exposure to patients, and ultimately decrease the risk of postoperative complications. 

## Conclusions

The newly introduced technique is straightforward, efficient, and effective in minimising operative time while instantaneously enhancing the accuracy of TAD estimation during cephalomedullary hip fracture stabilisation surgery. 

## References

[1] Schneider F, Geir F, Koidl C, Gehrer L, Runer A, Arora R (2023). Retrospective evaluation of radiological and clinical outcomes after surgical treatment of proximal femur fractures utilizing TFNA. Arch Orthop Trauma Surg.

[2] Baumgaertner MR, Curtin SL, Lindskog DM, Keggi JM (1995). The value of the tip-apex distance in predicting failure of fixation of peritrochanteric fractures of the hip. J Bone Joint Surg Am.

[3] Geller JA, Saifi C, Morrison TA, Macaulay W (2010). Tip-apex distance of intramedullary devices as a predictor of cut-out failure in the treatment of peritrochanteric elderly hip fractures. Int Orthop.

[4] Wijeratna MD (2014). A 'TAD' easier to calculate!. Ann R Coll Surg Engl.

[5] DePuy Synthes (2025). TFNA Advanced proximal femoral nailing system for intramedullary fixation of proximal femoral fractures: surgical technique. https://a490ebc3686a416702a4-875c9cad01df4876deaf64b6bbcf2205.ssl.cf2.rackcdn.com/ota_6d02298b40146f911e2f37490cd7f3fd.pdf.

[6] Haydar AJ, Hussainy Al (2005). "No high tech": a new, non-invasive technique for accurate guide wire placement in the dynamic hip screw fixation of femoral neck fractures. Eur J Orthop Surg Traumatol.

[7] (2025). Gamma3 hip fracture nailing system. https://www.stryker.com/us/en/trauma-and-extremities/products/gamma3.html.

[8] Smith & Nephew (2025). Smith & Nephew IMHS™ intramedullary hip screw: surgical technique. http://www.cambridgeorthopaedics.com/easytrauma/classification/surgtech/smithnephew/smithneph%20manuals/7118-0934_IMHS.pdf.

[9] (2025). Smith & Nephew Trigen Intertan intertrochanteric antegrade nail: surgical technique. https://smith-nephew-delivery.stylelabs.cloud/api/public/content/363921060e774c4ba2871bf028932340?v=b18ed469&amp;download=true.

[10] (2025). Aesculap® Targon® PFT intramedullary nail for proximal femoral fractures. Aesculap.

[11] Dupuy Synthes 119475-138107.pdf (2025). DHS/DCS system including LCP DHS and DHS blade: surgical technique. https://p1.aprimocdn.net/jjamp/en/depuy-synthes/ous-only-%E2%80%93-surgical-technique-guide-(stg)/119475-138107.pdf.

